# Exploring the perceived impact of pediatric simulations and pre-simulation activities in paramedic education: A quantitative post-intervention study

**DOI:** 10.5339/qmj.2025.86

**Published:** 2025-09-15

**Authors:** Anan Al Badawi, Guillaume Alinier, Stella Bosun Arije

**Affiliations:** 1 Department of Medical Education, Hamad Medical Corporation, Doha, Qatar; 2 Hamad Medical Corporation, Doha, Qatar; 3 Weill Cornell Medicine-Qatar, Doha, Qatar; 4 School of Health and Social Work, University of Hertfordshire, Hatfield, UK; 5 Faculty of Health and Life Sciences, Northumbria University, Newcastle upon Tyne, UK; 6 University of Doha for Science and Technology, Doha, Qatar *Email: aalbadawi1@hamad.qa

**Keywords:** Simulation-based learning, pre-simulation activities, paramedic education, pediatric emergencies, Qatar

## Abstract

**Background:**

Pediatric emergencies require specialized prehospital care due to children’s unique medical needs. This study explored the perceptions of a Middle Eastern Ambulance Service’s paramedics regarding the impact of pediatric-related simulation-based activities on their knowledge and confidence. It also evaluated the influence of pre-simulation activities, including online materials, lectures, video-based learning, and skills workstations, on their preparedness for these simulations.

**Methods:**

This quantitative study involved 225 paramedics who participated in a 1-day pediatric Continuous Professional Development program conducted between January and March 2022. Following the program, participants completed an online post-program survey utilizing the Simulation Effectiveness Tool-Modified (SET-M).

**Results:**

The SET-M results showed strong agreement (>90%) regarding the effectiveness of simulation in enhancing knowledge and confidence (M = 2.9, SD = 0.24–0.32). Participants also strongly agreed (>89.3) that pre-simulation activities improved their preparedness for simulation. Skills workstations received the highest ratings (M = 2.9, SD = 0.26), while video-based learning was slightly lower (M = 2.9, SD = 0.31). The breakdown analysis of two survey statements indicated that, regardless of experience, simulation exposure, or qualifications, participants positively perceived the effectiveness of both simulation scenarios and pre-simulation activities.

**Conclusion:**

The study highlights the paramedics’ perceived positive impact of simulation on their knowledge and confidence in managing pediatric emergencies, emphasizing the value of pre-simulation activities in enhancing their preparedness for the simulation-based activity. The findings hold practical implications for educators, curriculum designers, and paramedic practitioners in improving pediatric emergency training.

## 1. INTRODUCTION

Pediatric emergencies pose significant challenges for emergency medical services (EMS) providers, including the need for specialized equipment, tailored interventions due to children’s unique physiology, and the emotional toll on patients and caregivers.^1^ These emergencies may include conditions such as respiratory distress, seizures, anaphylaxis, cardiac arrest, and traumatic injuries. In the United States,36,288,405 EMS activations were reported in 2022, of which 2,152,849 (5.9%) were related to pediatric patients.^2^ Some of these pediatric emergencies required paramedics to perform advanced interventions such as medication calculation, airway management, and cardiopulmonary resuscitation.^2^ The proportion of adult to pediatric patients highlights the paramedics’ limited exposure to seriously ill or injured pediatric patients, as well as the insufficient opportunities for them to perform life-saving interventions.

Previous research has demonstrated that paramedics often lack adequate pediatric resuscitation skills due to insufficient relevant clinical exposure, inadequate training, anxiety, and skills that deteriorate over time.^3^ Cottrell *et a* ^4^ identified a range of factors related to paramedic practitioners, including limited exposure to pediatric cases, unfamiliarity with procedures such as airway management and immobilization, inadequate training, high anxiety when treating children due to a lack of confidence, and challenges in decision-making regarding interventions such as advanced airway techniques and intravenous cannulation.^2^ According to Hansen *et a*,^5^ these challenges can increase the likelihood of adverse safety events, medication errors, and paramedic practitioner burnout. This brings to the forefront the necessity of ensuring that paramedics receive sufficient preparation to effectively manage pediatric patients via other avenues.

Simulation-based learning (SBL) is an educational technique that replicates real experiences with guided, immersive scenarios designed to elicit or reproduce clinical experiences.^6^ This approach allows learners to practice newly acquired skills and concepts within a safe and controlled environment, promoting a more experiential form of education.^7^ SBL helps manage high-stakes pediatric emergencies by allowing paramedics to practice essential clinical skills, from vital sign assessment to life-saving interventions. SBL can enhance essential skills related to prehospital care, including technical skills, teamwork, effective communication, decision-making, and situational awareness as it provides a controlled and safe environment allowing paramedics to make mistakes, receive feedback, and refine their responses without compromising patient safety.^8^ Integrating SBL into paramedic education programs has become a key strategy to ensure paramedics are well-prepared and confident when confronted with prehospital emergencies involving children.^9^ One other aspect, the value of preparation before engaging in simulation-based learning activities to ensure a positive learning experience, should not be underestimated.^7,10^ This preparation may contain pre-briefing information so they understand what will be expected of them during the activity and what it may entail, as well as study material if it is shared with them well in advance.

In Qatar, the population demography is very skewed and includes a relatively low percentage of children, as it has a very large proportion of male migrant workers who reside in the country without their families.^11^ This further reduces the opportunities for Qatar’s EMS professionals to treat pediatric patients. Hence, SBL has an important role in getting paramedics to practice pediatric-specific skills. The Training Department of Hamad Medical Corporation Ambulance Service (HMCAS) has embedded SBL in most of its courses to ensure clinical staff can have hands-on practice (N = 1,660 in early 2022) in a safe and controlled environment. It is even part of the formative and summative assessment sessions organized in the form of Objective Structured Clinical Examination sessions in various courses, some of which include pediatric-specific stations.^12,13^ A 2019 study found that a pediatric Continuous Professional Development (CPD) course improved the knowledge of HMCAS paramedics. Participants perceived the simulations and pre-simulation activities as helpful in enhancing their pediatric patient assessment and management skills. However, the study did not examine the role of pre-simulation activities in preparing participants for simulation.^14^

Simulation pre-briefing encompasses all events before the start of a simulation scenario, including preparing learners for the educational content to be covered in the simulation-based activity and establishing the rules for the simulation experience (preparation), and familiarizing them with the simulation environment and equipment (orientation).^15^ Then, before each scenario, there is a short briefing to set the scene and potentially assign specific roles to each participant (e.g., “Consider that this case takes place early morning on a construction site with participant X as the primary clinician. This is the dispatch information you received: …”). The pre-simulation preparation aims to equip the participants with the necessary knowledge and skills for the simulation experience.^16^ This may include conducting pre-simulation activities such as presentations, skills workshops, quizzes, and group discussions, and orienting learners to the environment and equipment.^17^ Current literature on effective SBL in paramedic education focuses on scenarios and debriefing, with limited attention to the preparatory phase for learners.

This study aimed to investigate HMCAS staff perceptions of the impact of the SBL components of pediatric CPD programs on their knowledge and confidence in handling pediatric emergencies, and also to determine how they thought the pre-simulation activities impacted their preparedness for taking part in the scenarios.

## 2. METHODS

This quantitative, post-educational intervention survey-based study utilized a convenience sample of paramedics who attended a one-day, face-to-face pediatric CPD training program (Appendix A). This 3-month program was delivered multiple times by the HMCAS Training Department over 3 months (January to March 2022) and followed by a post-course survey based on the Simulation Effectiveness Tool-Modified (SET-M).^18^

SET-M is a 19-item survey that has been validated and is used to evaluate participants’ perceptions of the effectiveness of simulation-based experiences in meeting their learning needs and comprises four subscales (pre-briefing: 2 items; learning: 6 items; confidence: 6 items; debriefing: 5 items).^18^ To the best of the authors’ knowledge, this is the first study to explore the perceived effectiveness of simulation among paramedics using the SET-M. However, the tool has not undergone a validity testing process due to the overlap in the paramedic and nursing scopes of practice, particularly in patient assessment, basic medical procedures, and emergency care.^19^

The survey was modified to complete the original tool by collecting additional demographic information about the participants (age, gender, nationality, professional scope, education, years of experience at HMCAS, and prior exposure to SBL sessions) as well as their perceptions concerning the pre-simulation activities and how they impacted their readiness for the simulation-based activities. These questions were developed by reviewing previous research on similar topics and the Healthcare Simulation Standards of Best Practice,^10,20^ as there is increasing evidence of the role played by pre-simulation activities in enhancing participants’ essential knowledge and skills. Three key areas for investigation were identified: whether the pre-simulation activities enhanced participants’ confidence, encouraged active participation in the simulation, and improved engagement in the subsequent debriefing sessions. A three-point Likert scale was adopted to facilitate the data analysis process (1 = Do Not Agree, 2 = Somewhat Agree, 3 = Strongly Agree), aligning with the scale used for the SET-M. Subsequently, the researcher collaborated with the pediatric CPD team to review and refine the questions, and a draft was created. Five paramedics were randomly asked to review them and provide feedback regarding clarity, relevance, and ease of understanding. They were requested not to participate in the study if they attended that CPD activity during the data collection period. The feedback was incorporated, and revisions were made accordingly. The questions were finalized in collaboration with the pediatric CPD team.

### 2.1 Study Participants

The inclusion criteria of the study sample were employment as an Ambulance Paramedic (AP) or Critical Care Paramedic (CCP) within HMCAS and participation in the pediatric CPD training program during the study period. The HMCAS emergency response system comprised 1,557 APs and 104 CCPs operating in a two-tier model.^21^ APs perform tasks such as defibrillation, supraglottic airway intubation, and medication administration, while CCPs manage advanced procedures like endotracheal intubation, ventilator setup, and sedation. Both roles require a bachelor’s degree in paramedicine or nursing, three years of experience in emergency or prehospital care, and completion of a local training program involving over 40 simulation sessions using diverse modalities.^21^ APs respond to all emergencies, with CCPs engaged only in critical cases. To ensure learning relevance in the pediatric CPD program, participants were assigned scenario roles aligned with their professional scope of practice.

### 2.2 Sample Size

The sample size for this study was calculated using https://www.calculator.net/sample-size-calculator.html, targeting 309 APs and 83 CCPs with a 95% confidence level. The Scheduling Department planned participant attendance, which minimized selection bias by the authors.

### 2.3 Pre-Simulation Activities and Their Significance

The pre-simulation activities are outlined in Appendix B The online materials need to be completed a week before the participants attend the CPD activity in the training venue, while the other pre-simulation activities are done on the CPD day. They are structured to ensure candidates are well-prepared for the simulation component and capable of applying their skills to effectively manage pediatric emergencies.

### 2.4 Simulation Component

The simulation component of the pediatric CPD program includes three scenarios selected for their relevance to common and critical pediatric emergencies encountered in prehospital care (see Appendix C). These scenarios aim to develop essential resuscitation skills, critical thinking, and teamwork to improve patient safety and outcomes. The scenarios were designed following the INACSL Standards of Best Practice—Healthcare Simulation Standards of Best Practice (HSSOBP) and reviewed by the paramedic CPD instructors for validity. They were facilitated in a way that provided total autonomy to the participants and were each followed by a debriefing session using the Plus Delta model.^22^ The debriefers were the paramedic CPD instructors who hold at least five years of teaching experience using simulations and receive their training at the Itqan Clinical Simulation and Innovation Center in Qatar.

### 2.5 Data Collection

At the conclusion of each workshop, two non-teaching colleagues facilitated the data collection. A QR code was provided to those who agreed to participate and access an anonymous survey hosted on Microsoft Forms on their mobile phones. The survey link was also sent to participants’ work email accounts as an alternative access method. This study was accepted by the Ambulance Service Group Research Oversight Community as a service improvement study (AS2022-630). Participants were explicitly informed that their participation was entirely voluntary and that completing the survey was considered consent to participate.

### 2.6 Data Analysis

The study used a content-driven approach in writing the results section and the method of analysis in content analysis. Content analysis is a research technique used to determine the presence of certain words, themes, or concepts within some given qualitative and quantitative data. It allows for both qualitative and quantitative analysis.^23^

## 3. RESULTS

### 3.1 Demographic Characteristics

Due to logistical constraints, the pediatric program, offered 7 to 8 times per month for up to 18 participants, resulted in a final sample of 225 participants (181 APs and 44 CCPs) who provided consent. While the sample size was significant, it fell short of targets, achieving confidence intervals of 85% for APs and nearly 50% for CCPs. Demographics are summarized in Table 1. Participants’ ages ranged from 20 to >45 years (M = 36, SD = 8.35), with most aged 20 to 35 years (44.9%). Males comprised 88.4% of the sample, and 44.4% had 1 to 5 years of experience at HMCAS. APs and CCPs represented 80.4% and 19.6%, respectively. Regarding experience, 34.2% had 6 to 10 years, 12.9% over 15 years, and 8.4% had 11 to 15 years.

Most participants held bachelor’s degrees (71.6%), while diplomas and postgraduate qualifications were held by 22.2% and 6.2%, respectively. Simulation exposure ranged from 10.2% for those with over 15 years of experience to 44.9% for those with 1 to 5 years. Participants from the North Africa region accounted for the highest proportion at 22.6%, while those from Western regions represented the lowest at 5.3%. Regional diversity can influence communication styles and learning experiences by shaping participants’ cultural norms, language preferences, and approaches to teamwork and decision-making, all of which may impact how they engage in and benefit from simulation-based education.

### 3.2 The Perceived Preparedness for the Simulation Experience

Table 2 shows the perceived impact of pre-simulation activities on participants’ preparedness for the simulation component. Responses across the Participation in Simulation, Confidence to Participate in Simulation, and Participation in Debriefing Sessions subscales indicated strong agreement, ranging from 89.3% to 93.8%. Mean scores (M = 2.9, SD = 0.24–0.32) reflected positive perceptions of the pre-simulation activities. Two themes emerged from the first data set regarding paramedics’ preparedness for the simulation.

1. Optimal confidence, readiness, and preparedness for knowledge acquisition are key.
2. Diverse workshop resources promote high readiness, confidence, and participation.

### 3.3 The Perceived Effectiveness of SBL in Enhancing Knowledge and Confidence 

The participants’ responses to the SET-M are summarized in Table 3. For the pre-briefing subscale, strong agreement ranged from 89.3% to 92.4%, with (M = 2.8–2.9, SD = 0.26–0.31). Across the learning and confidence subscales, strong agreement ranged from 91.6% to 93.6% (M = 2.9, SD = 0.25–0.3). The debriefing subscale showed even stronger agreement, with 90.7% to 95.1% strongly agreeing (M = 2.9, SD = 0.22–0.30).

However, the second set of data produces two themes related to the perceived effectiveness of SBL and CPD in enhancing the knowledge and confidence of paramedics.

1. Simulation debriefing is a key tool for improving learners’ knowledge.
2. Training and constructive feedback promote sustained confidence and team performance.

### 3.4 Preferences Breakdown According to Sociodemographic

Table 4 shows the impact of the statement, “I am more confident in providing interventions that foster patient safety.” Most participants strongly agreed that the scenarios positively impacted their confidence (79.3%–97%).

Table 5 illustrates the impact of skills workstations on participants’ confidence in participating in the simulation-based activity. Most participants strongly agreed that the skills stations effectively enhanced their confidence (87%–100%).

However, the third set of data revealed two themes that depict the participants’ demographics in relation to the perceived impacts of the scenarios and pre-simulation activities.

1. Using various scenarios promotes clinical confidence for safe patient care delivery.
2. Simulation training and multiple workshops are upskilling tools for all categories of paramedics.

## 4. DISCUSSION

Pediatric-related CPD programs in Qatar are limited.^24^ Evaluating learners’ experiences helps identify areas for improvement, enhancing program effectiveness.^25^ Research shows that learner satisfaction boosts self-confidence, facilitating skill development and knowledge acquisition. Satisfaction with simulations, as noted by Mayville,^26^ is linked to improved clinical reasoning and knowledge consolidation. Hence, integrating pediatric-specific simulations into CPD programs can give paramedics the practical knowledge and confidence needed for effective pediatric prehospital care.

Many studies have confirmed that SBL can enhance paramedics’ abilities in managing pediatric emergencies. Studies highlight its effectiveness in improving clinical decision-making during mass casualty triage^27^ and replicating challenging scenarios for training.^28^ An additional systematic review revealed that randomized studies showed improved team performance in re-evaluations of neonatal resuscitation simulations 3 to 6 months after the training.^29^

The results indicate that participants found the three scenarios effective in enhancing their knowledge and confidence in managing pediatric emergencies. Analysis of SET-M items, particularly the learning and confidence subscales, supported these findings. Participants also highlighted the positive impact of pre-simulation activities on their preparedness for the simulation experience.

### 4.1 Participants’ Perceptions of Simulation’s Impact on Knowledge and Confidence

Leighton *et a* ^18^ noted the absence of a standardized method for scoring the SET-M and recommended focusing on areas with lower agreement. While this study showed strong overall agreement with SET-M items, disagreements on two scenario subscale questions suggest minor concerns about scenario complexity, realism, or clinical accuracy, which may affect confidence and decision-making. These findings align with prior research showing that simulation-based training improves paramedics’ knowledge and confidence in managing pediatric emergencies.^30–32^

***4.1.1 Pre-briefing Subscale***

Most participants strongly agreed on the positive impact of pre-briefing on their learning and confidence (Table 3). These findings emphasize the importance of pre-briefing in creating a safe learning environment, fostering psychological safety, and encouraging active engagement. This aligns with^33^ and the HSSOBP, which highlights pre-briefing as essential for preparing learners and conveying key information about the simulation.^10^ Similarly, Chamberlain^34^ found that pre-briefing positively influenced nursing students’ perceptions of simulation effectiveness, learning, and confidence.

***4.1.2 Learning and Confidence Subscales***

Participants strongly agreed with the positive impact of simulation training on their knowledge and confidence (Table 3). These findings align with Glomb *et a*,^31^ who reported that simulation training enhanced pediatric knowledge, self-confidence, and skills examination scores. Similarly, Farrell *et a* ^35^ found that pediatric simulation curricula improved knowledge and confidence in caring for pediatric patients. Stopyra *et a* ^32^ also highlighted increased confidence and comfort in managing airway emergencies and performing pediatric needle cricothyrotomy.

The scenarios in this study were designed following the HSSOBP for Simulation Design and Facilitation Methods,^36,37^ creating an evidence-based learning experience that fostered active engagement and skill development. The realistic settings of the scenarios supported the development of problem-solving, decision-making, critical thinking, and communication skills, consistent with findings by Marshall *et a* ^38^ and Rossler *et a*.^20^

***4.1.3 Debriefing Subscale***

The debriefing subscale revealed strong agreement (Table 3), highlighting the effectiveness of the debriefing sessions in creating a supportive learning environment and meeting participants’ educational needs, consistent with findings by Ahmad *et a* ^39^ and Guerrero *et a*.^40^ The positive outcomes can be attributed to skilled facilitation, which fostered participant engagement during debriefing, as noted by Abulebda *et a*.^41^

### 4.2 Participants’ Preparation for the Simulation Component

To the best of our knowledge, this study is the first to evaluate the perceived impact of pre-simulation activities on paramedics’ preparedness for simulation experiences. Effective pre-simulation preparation is crucial for optimizing the SBL experience and reducing the learners’ cognitive load and anxiety.^10^ Clear instructions and pre-simulation activities have been shown to improve learner performance.^42^ Adequate preparation, including orientation and scenario exposure, helps create a supportive environment, reducing anxiety and promoting critical thinking and reflective practice.^43^

In this study, most participants reported that the pre-simulation activities improved their performance during scenarios, increased their confidence, and enhanced their engagement in the debriefing sessions (Table 2). Online learning materials were perceived as highly effective, probably due to their accessibility and flexibility, allowing participants to review the content at their own pace, as noted by Song *et a*.^44^ Interactive elements likely improved their understanding and boosted their confidence for the debriefing discussions, as pointed out by Skulmowski *et a*.^45^ Similarly, as recommended by Gordani *et a*,^46^ PowerPoint presentations were well-received, with their structured and visually engaging design facilitating comprehension and retention of key concepts.^47^ Case-based discussions within the presentations further reinforced learning, especially in pediatric emergencies.

In this study, considering the “somewhat agree” responses as partial agreement, skill workstations were identified as the most effective pre-simulation activities, with strong agreement ranging from 92.9% to 93.8%. This suggests that hands-on practice plays a vital role in skill development and confidence-building, which is important for simulation readiness,^48,49^ and Lammers *et a*.^46^ Video-based learning (VBL) was also deemed to be highly effective (strong agreement ranging from 89.3% to 90.2% but to a lesser degree than the other pre-simulation activities.

The results highlight the value of incorporating diverse pre-simulation activities to cater to varied learning preferences and styles, including visual, auditory, and kinesthetic modalities.^50^ Offering a combination of activities—such as online modules, presentations, VBL, and hands-on stations—ensures inclusivity, optimizes participant engagement, and enhances preparedness for simulation experiences.

### 4.3 The Content Analysis for Both Selected Statements

High agreement across all preferences suggests that the program effectively met participants’ needs. Even among those with minimal HMCAS work experience or prior simulation exposure, the simulation component was perceived as valuable, with minimal disagreement observed across all categories (Tables 4,5). These findings indicate that participants believed the program enhanced their knowledge and confidence in managing pediatric emergencies and facilitated meaningful engagement with the simulation-based experience. These results contribute to the study’s overarching objectives by demonstrating that the CPD program, regardless of participants’ backgrounds, effectively supports learning and simulation preparedness.

### 4.4 Limitations

One limitation is that the survey was administered late in the day, potentially leading participants to rush when completing it. To minimize these biases, the study was introduced early to potential participants. Future studies may include follow-up assessments to evaluate perceived knowledge retention and longer-term impact.

The study was conducted within a single institution, limiting the generalizability of the findings. While the sample size was reasonable, it did not reach the target of 309 participants, likely due to the program’s short duration and limited workshop offerings. However, the study involved a diverse group of paramedics in a high-volume EMS system that can potentially increase the generalizability of the findings within similar clinical and educational settings.

The cross-sectional design limited the in-depth analysis of participants’ responses. However, the study used a validated evaluation tool (SET-M) alongside a structured scenario-based approach tailored to the participants’ scope of practice. This design enhanced the relevance and realism of the learning experience and contributed to the reliability of the data collected. While results showed strong agreement on the benefits of pre-simulation and simulation activities, the study focused solely on perceived improvements. The results didn’t verify participants’ competence in providing effective care for critically ill pediatric patients.

## 5. CONCLUSION

This study assessed the perceived effectiveness of a simulation-based CPD program in enhancing paramedics’ knowledge and confidence in managing pediatric emergencies, as well as the role of pre-simulation activities in preparing participants for simulation. The findings suggest that, regardless of participants’ backgrounds, the simulation component was generally seen as effective in enhancing their knowledge and confidence, with few disagreeing responses. While some concerns were noted regarding clinical decision-making and safe interventions, these responses were too few to warrant changes to the simulation design.

Additionally, pre-simulation activities were found to enhance participants’ readiness to take part in the scenarios, highlighting the effectiveness of a multimodal approach to pre-simulation activities in meeting diverse learning needs and enhancing the simulation experience. Future iterations can integrate objective assessments to measure skill development and ensure real-world application. The results can help paramedic educators refine simulation design and pre-simulation activities to enhance learning outcomes.

## LIST OF ABBREVIATIONS

**Table:** 

AP	Ambulance Paramedic
CCP	Critical Care Paramedic
CPD	Continuous Professional Development
EMS	Emergency Medical Services
HMCAS	Hamad Medical Corporation Ambulance Service
HSSOBP	Healthcare Simulation Standards of Best Practice
SBL	Simulation-based learning
SET-M	Simulation Effectiveness Tool – Modified
VBL	Video-based learning

## Figures and Tables

**Table 1.** Socio-demographic characteristics of the study participants.

Demographic variables	*N* = 225	
**Age in years (M = 36: SD = 8.35)**	*n*	**%**
20–35	101	44.9
36–45	93	41.3
>45	31	13.8
**Gender**	*n*	**%**
Male	199	88.4
Female	26	11.6
**Scope of practice**	*n*	**%**
Ambulance Paramedic	181	80.4
Critical Care Paramedic	44	19.6
**Experience in HMCAS**	*n*	**%**
1–5 years	100	44.4
6–10 years	77	34.2
11–15 years	19	8.4
>15 years	29	12.9
**Educational level**	*n*	**%**
Diploma	50	22.2
Bachelor	161	71.6
Postgraduate	14	6.2
**Exposure to simulation**	*n*	**%**
1–5 years	101	44.9
6–10 years	79	35.1
11–15 years	22	9.8
>15 years	23	10.2
**Nationalities**	*n*	**%**
North Africa (Algeria, Egypt, Tunisia)	51	22.6%
Middle East (Iraq, Jordan, Syria, Yemen)	47	20.9%
South Asia (India)	42	18.8%
Southeast Asia (Philippines)	40	17.8%
Sub-Saharan Africa (South Africa, South Sudan)	33	14.6%
Western Countries (Canada, the United Kingdom, the United States of America)	12	5.3%

**Table 2.** The perceived impact of the pre-simulation activities on the preparedness for the simulation-based activities (*N* = 225).

Subscale and statement	Do not agree		Somewhat agree		Strongly agree		Mean/SD	
Participation in simulation	*n*	%	*n*	%	*n*	%	M	SD
Online materials	0	0	15	6.7	210	93.3	2.9	0.25
PowerPoint presentations	0	0	14	6.2	211	93.8	2.9	0.24
Video-based learning	0	0	24	10.7	201	89.3	2.9	0.31
Skills workstation	0	0	16	7.1	209	92.9	2.9	0.26
Confidence to participate in the simulation	*n*	%	*n*	%	*n*	%	M	SD
Online materials	1	0.4	18	8	206	91.6	2.9	0.30
PowerPoint presentations	1	0.4	21	9.3	203	90.2	2.9	0.32
Video-based learning	0	0	22	9.8	203	90.2	2.9	0.30
Skills workstation	1	0.4	13	5.8	211	93.8	2.9	0.27
Participation in the debriefing sessions	*n*	%	*n*	%	*n*	%	M	SD
Online materials	0	0	14	6.2	211	93.8	2.9	0.24
PowerPoint presentations	1	0.4	17	7.6	207	92.0	2.9	0.29
Video-based learning	0	0	24	10.7	201	89.3	2.9	0.30
Skills workstation	0	0	16	7.1	209	92.9	2.9	0.31

**Table 3.** Participants’ response to the Simulation Effectiveness Tool-Modified (SET-M; *N* = 225).

Subscale and statements	Do not agree		Somewhat agree		Strongly agree		Mean/SD	
**Pre-briefing (2 items)**	***n***	**%**	***n***	**%**	***n***	**%**	**M**	**SD**
Pre-briefing increased my confidence.	0	0	17	7.6	208	92.4	2.9	0.26
Pre-briefing was beneficial to my learning.	0	0	24	10.7	201	89.3	2.8	0.31
**Learning subscale (6 items)**	***n***	**%**	***n***	**%**	***n***	**%**	**M**	**SD**
I am better prepared to respond to changes in my patient’s condition.	0	0	15	6.7	210	93.3	2.9	0.25
I developed a better understanding of the pathophysiology.	0	0	19	8.4	206	91.6	2.9	0.28
I am more confident in my assessment skills.	0	0	15	6.7	210	93.3	2.9	0.25
I felt empowered to make clinical decisions.	1	0.4	18	8.0	206	93.6	2.9	0.30
I developed a better understanding of medications.	0	0	18	8.0	207	92	2.9	0.27
I had the opportunity to practice my clinical decision-making skills.	0	0	17	7.6	208	92.4	2.9	0.26
**Confidence subscale (6 items)**	***n***	**%**	***n***	**%**	***n***	**%**	**M**	**SD**
I am more confident in my ability to prioritize care and interventions	0	0	16	7.1	209	92.9	2.9	0.25
I am more confident in communicating with my patients.	0	0	20	8.9	205	93.3	2.9	0.28
I am more confident in my ability to teach patients about their illness and interventions.	0	0	19	8.4	206	91.6	2.9	0.27
I am more confident in my ability to report information to the healthcare team.	0	0	17	7.6	208	92.4	2.9	0.26
I am more confident in providing interventions that foster patient safety.	1	0.4	18	8	206	91.6	2.9	0.30
I am more confident in using evidence-based practice to provide care.	0	0	18	8	207	92	2.9	0.27
**Debriefing subscale (5 items)**	***n***	**%**	***n***	**%**	***n***	**%**	**M**	**SD**
Debriefing contributed to my learning.	0	0	11	4.9	214	95.1	2.9	0.22
Debriefing allowed me to communicate my feelings before focusing on the scenario.	0	0	20	8.8	205	91.1	2.9	0.28
Debriefing was valuable in helping me improve my clinical judgment.	0	0	19	8.4	206	91.6	2.9	0.28
Debriefing provided opportunities to reflect on my performance during the simulation.	0	0	21	9.3	204	90.7	2.9	0.30
Debriefing was a constructive evaluation of the simulation.	0	0	18	8	207	92	2.9	0.27

**Table 4.** Participants’ confidence in providing patient safety interventions.

Group	Category	Do not agree (*n*/%)	Somewhat agree (*n*/%)	Strongly agree (*n*/%)	Total sample (*N*/%)
Years of HMCAS work experience	1–5 years	0 (0%)	3 (2.1%)	97 (97%)	100 (44.4%)
	6–10 years	1 (1.3%)	6 (7.8%)	70 (90.9%)	77 (34.2%)
	11–15 years	0 (0%)	3 (15.8%)	16 (84.2%)	19 (8.4%)
	>15 years	0 (0%)	6 (20.7%)	23 (79.3%)	29 (12.9%)
Simulation exposure	1–5 years	1 (1%)	3 (3%)	97 (96%)	101 (44.9%)
	6–10 years	0 (0%)	8 (10.1%)	71 (89.9%)	79 (35.1%)
	11–15 years	0 (0%)	2 (9.1%)	20 (90.9%)	22 (9.8%)
	>15 years	0 (0%)	5 (21.8%)	18 (78.2%)	23 (10.2%)
Educational level	Diploma	0 (0%)	9 (10.7%)	75 (89.3%)	84 (37.3%)
	Bachelor	1 (0.8%)	8 (6.5%)	115 (92.7%)	124 (55.1%)
	Postgraduate	0 (0%)	1 (5.9%)	16 (94.1%)	17 (7.6%)

**Table 5.** Perceived impact of skills workstations.

Group	Category	Do not agree (*n*/%)	Somewhat agree (*n*/%)	Strongly agree (*n*/%)	Total sample (*N*/%)
Years of experience	1–5 years	0 (0.0)	2 (2.0)	98 (98.0)	100 (44.4)
	6–10 years	1 (1.3)	6 (7.8)	70 (90.9)	77 (34.2)
	11–15 years	0 (0.0)	2 (10.5)	17 (89.5)	19 (8.4)
	>15 years	0 (0.0)	3 (10.3)	26 (89.7)	29 (12.9)
Simulation exposure	1–5 years	1 (1.0)	3 (3.0)	97 (96.0)	101 (44.9)
	6–10 years	0 (0.0)	5 (6.3)	74 (93.7)	79 (35.1)
	11–15 years	0 (0.0)	2 (9.1)	20 (90.9)	22 (9.8)
	>15 years	0 (0.0)	3 (13.0)	20 (87.0)	23 (10.2)
Educational level	Diploma	0 (0.0)	5 (6.0)	79 (94.0)	84 (37.3)
	Bachelor	1 (0.8)	8 (6.5)	115 (92.7)	124 (55.1)
	Postgraduate	0 (0.0)	0 (0.0)	17 (100.0)	17 (7.6)

## Appendix

**APPENDIX A:** Timetable of the Hamad Medical Corporation Ambulance Service pediatric CPD training program.

Time	Activity
6:45–7:00	Welcome and Registration
7:00–8:00	Session 1: Presentation—Pediatric cardiac arrest
8:00–8:20	Session 2: Video-based learning—Pediatric assessment triangle
8:20–8:30	Coffee break
8:30–09:30	Session 3 Presentation—Management of pediatric emergencies
09:30–11:30	Session 4: Skills workshop
11:30–12:30	Lunch break
12:30–15:15	Session 5: Three Simulation scenarios with debriefing
15:15–15:30	Program summary—Question and answer session
15:30–15:40	Activity evaluation and signing out

## Appendix

**APPENDIX B:** Pre-simulation activities for the pediatric CPD course and their significance.

Activity	Content	Expected duration	Significance
Online learning materials	Participants access three interactive modules on pediatric cardiac arrest, respiratory, and trauma emergencies a week before the program. The online materials must be finished along with a pre-course quiz to qualify for the face-to-face part of the CPD session.	180 minutes	Ensures candidates are adequately prepared, addresses critical emergency topics, and enhances participants’ readiness for the simulation-based experience
PowerPoint presentation	Topics from the online materials are presented as case studies to engage participants during the CPD course and encourage critical thinking.	105 minutes	Deepens engagement, improves critical thinking, and prepares participants to apply knowledge in practical scenarios.
Video-based learning	Participants are shown two videos showcasing pediatric respiratory distress, followed by guided discussions on symptom recognition and immediate interventions.	20 minutes	Improves the ability to identify and respond effectively to pediatric respiratory emergencies, enhancing participants’ clinical decision-making.
Practical skill stations	Three stations focus on pediatric airway skills, initial cardiac arrest management, and multiple skills like drug administration and nebulization.	120 minutes	Enhances proficiency in essential skills and provides practical experience to reduce cognitive load during simulation activities.

## Appendix

**APPENDIX C:** Scenarios and learning objectives in the pediatric CPD program.

Scenario title	Description	Learning objectives
Anaphylactic reaction	A 6-year-old child is experiencing a severe anaphylactic reaction requiring prompt identification and treatment.	•Identify and treat anaphylactic reactions in line with HMCAS clinical practice guidelines. •Demonstrate effective team communication. •Engage empathetically with child and parents while providing clear and accurate information.
Life-threatening asthma	A 4-year-old child suffering from a severe asthma attack progressing to cardiopulmonary arrest, requiring CPR and ROSC management.	•Recognize respiratory distress in asthma. •Manage bradycardia and cardiac arrest during asthma exacerbation. •Perform high-quality CPR and post-resuscitation care following HMCAS clinical practice guidelines.
Traumatic arrest	A 9-year-old child in cardiac arrest following trauma, necessitating advanced life support interventions and resuscitation efforts.	•Recognize and manage traumatic cardiac arrest. •Perform high-quality compressions and advanced life support, including intraosseous access and defibrillation. •Execute post-ROSC care effectively for improved patient outcomes.

HMCAS, Hamad medical corporation ambulance service; CPR, Cardiopulmonary resuscitation; ROSC, Return of spontaneous circulation.
